# Is there still a role for systematic biopsy after targeted biopsy for the detection of clinically significant prostate cancer in MRI suspicious lesions?

**DOI:** 10.1590/S1677-5538.IBJU.2025.0653

**Published:** 2026-02-28

**Authors:** João M. Pina, João Guerra, Miguel B. Lança, João L. Dias, Rita N. Lucas, Luis C. Pinheiro

**Affiliations:** 1 ULS São José Departamento de Urologia Lisboa Portugal Departamento de Urologia, ULS São José, Lisboa, Portugal; 2 Hospital Cruz Vermelha Departamento de Urologia Lisboa Portugal Departamento de Urologia, Hospital Cruz Vermelha, Lisboa, Portugal; 3 Hospital Lusíadas Lisboa Departamento de Radiologia Lisboa Portugal Departamento de Radiologia, Hospital Lusíadas Lisboa, Lisboa, Portugal

**Keywords:** Prostatic Neoplasms, Biopsy, Magnetic Resonance Imaging

## Abstract

**Purpose:**

The combination of systematic biopsy (SB) and MRI-targeted biopsy (TB) is the current approach for prostate cancer (PCa) diagnosis; however, the clinical benefit of including SB remains controversial. This study aimed to determine whether SB adds value beyond TB in detecting clinically significant prostate cancer (csPCa) in men with suspicious lesions.

**Materials and Methods:**

Retrospective, single-center study conducted between January 2019 and September 2023. It enrolled men with suspicious lesions identified on multiparametric MRI (PI-RADS≥3) who had undergone combined biopsy (TB+SB). Sociodemographic and clinical data were secondarily collected. csPCa was defined when ISUP≥2.

**Results:**

This study included 997 men with a median age of 68 years, of whom 497 had a negative prior biopsy. The TB+SB approach identified 53.0% of PCa and 36.8% of csPCa cases. TB alone significantly outperformed SB in identifying csPCa, with detection rates of 34.8% vs. 10.3%, respectively, missing only 4.8% of PCa diagnosis—most of which were low-grade tumors. SB contributed marginally, identifying additional csPCa cases in 1.4% of patients. In patients with a prior negative biopsy, the addition of SB to TB only accounted for 12.5% of PCa diagnosis. Limitations include the study single-center design, restricting generalizability, and the lack of whole-mount prostatectomy for histological confirmation.

**Conclusions:**

In conclusion, SB adds limited diagnostic value, with TB alone being sufficient for detecting csPCa cases in patients with MRI-visible lesions. The results suggest that SB may be safely omitted in selected patients to reduce biopsy burden and lead to better clinical outcomes.

## INTRODUCTION

Prostate cancer (PCa) is a prevalent malignancy, ranking second as the most common cancer in men (1). One to three million cases of prostate cancer are diagnosed every year (2). Incidence varies greatly from 6.3 to 83.4 per 100,000 men worldwide, with Europe and North America presenting some of the highest rates (3). In Portugal, there were 62.6 PCa new cases and 11.1 related deaths per 100,000 individuals in 2022. These numbers are expected to rise up to 22% and 54%, respectively, by 2045 in the Portuguese population (3).

Blood tests for prostate-specific antigen (PSA) and digital rectal examination continue to be the main methods for initial screening and evaluation of PCa (4). Transrectal ultrasound (TRUS)-guided systematic biopsy (SB) has been the preferred diagnostic approach for PCa since a pivotal 1989 study demonstrated its superiority over digitally directed biopsy sampling (5). Currently, the gold standard diagnostic tool is the 12-core extended sextant TRUS. However, its essentially random needle placement leads to a high false-negative rate and can underestimate tumor grade in up to 38% of cases due to undersampling (6). On the other hand, random TRUS biopsy frequently detects low-grade, indolent cancers, potentially leading to overtreatment. Consequently, there is a growing need to improve patient selection and enhance biopsy techniques to better identify and target potentially aggressive lesions.

Multiparametric magnetic resonance imaging (mpMRI) has revolutionized the diagnosis and local staging of PCa. Suspicious lesions identified using Prostate Imaging-Reporting and Data System (PI-RADS) v2.1 criteria (7), can be targeted and fused with real-time ultrasound for biopsy (8). MRI/TRUS fusion, also known as targeted biopsy (TB), has improved the detection of clinically significant prostate cancer (csPCa) by enabling a more effective sampling of prostate lesions (9). Despite its effectiveness, the 2024 European Association of Urology Guidelines advise that biopsy-naive men should still undergo both SB of 12 prostate regions and TB to minimize the risk of missing csPCa. For men with a prior biopsy, SB may be omitted, although weakly recommended (10).

Notwithstanding, the added diagnostic value of combining SB with TB remains a matter of ongoing debate. We hypothesize that SB does not provide added value in the context of the diagnosis of clinically significant PCa. In this context, this study aimed to evaluate whether a random systematic approach still provides additional value beyond fusion-guided biopsies in detecting csPCa in men with suspicious lesions identified on mpMRI.

## MATERIALS AND METHODS

### Study Participants and Design

This is a retrospective study conducted in a tertiary care center between January 2019 and September 2023, including men with clinical suspicion of PCa, who were biopsy-naïve or had a previous negative SB, who underwent a mpMRI with at least one suspicious lesion (PI-RADS≥3) identified and had both TB and SB. Patients were excluded based on the following criteria: previous treatment for prostate cancer, absence of MRI-visible prostate lesions, or an inability to undergo MRI. All the patients provided written informed consent.

### Data collection

Data were secondarily collected from the hospital medical records. It included sociodemographic characteristics (age) and clinical information (PSA levels, prostatic volume, lesions characteristics, PI-RADS and ISUP scores).

### Image Acquisition and Analysis

mpMRI images were obtained using 1.5 and 3-Tesla systems without using an endorectal coil following PIRADS v2.1 guidelines (11). Examination included T2-weighted (T2WI) images in three planes and functional sequences (DWI, ADC map and DCE) (12). Images were interpreted by experienced uro-radiologists, who segmented and marked up to two suspicious lesions using dedicated software (MiM-Symphony®), prioritizing those with the highest scores. Scans with inadequate image quality were repeated before biopsy.

### Combined biopsy procedure and evaluation

All biopsies were done under general anesthesia by the same experienced urologist. Patients initially underwent TB followed by SB using the transperineal approach (13). The perineum was sterilized and mpMRI-TRUS software (MiM-Symphony®) was used to guide transperineal TB using a template grid, with a median of five cores taken per lesion. This was followed by systematic biopsy of the entire prostate, with denser sampling of the peripheral zone (Supplementary Figure-1). Core locations were precisely tracked and documented using a 3D prostate model with automatic motion compensation, and biopsy sites were visualized on MRI through image fusion. Samples were labeled by location and analyzed by experienced uropathologists according to ISUP grading. Clinically significant prostate cancer was defined as ISUP grade group ≥2, as ISUP 1 is not considered to be clinically significant, and patients do not require treatment. Cancer detection rates were compared between targeted biopsy, systematic biopsy alone, and their combination.

## Statistical Analysis

All variables were summarized using descriptive statistics. Numerical variables were reported as medians with first and third quartiles (P25; P75), while categorical variables were described using absolute and relative frequencies. Demographic and clinical characteristics were analyzed for the overall sample and by prior biopsy history. PI-RADS score distributions were summarized, and prostate cancer detection rates were compared between systematic and targeted biopsies using McNemar's tests. Associations between biopsy methods and cancer detection were also assessed with McNemar's tests. Relationships between prior biopsy history and categorical variables were evaluated using Chi-squared or Fisher's exact tests, as appropriate. PI-RADS and ISUP scores were summarized by biopsy type, and ISUP score agreement between systematic and targeted biopsies was assessed with Cohen's kappa. All inferential tests were two-tailed, with a significance threshold of 5%.

The number needed for diagnosis (NND) was calculated as the inverse of the Youden index. Confidence intervals and comparisons between diagnostic methods were obtained using non-parametric bootstrap resampling (2,000 iterations), with statistical significance defined by a 95% confidence interval excluding zero.

A multinomial logistic regression analysis was performed to assess factors associated with patterns of prostate cancer detection. SB only was used as the reference category. Predictors included age, PSA, prostate volume and PSA density. Odds ratios (OR) and 95% confidence intervals (CI) were calculated for each predictor. Categories with small sample sizes were interpreted qualitatively if estimates were unstable. Patients without prostate cancer were excluded from this analysis.

The statistical analysis was carried out using the RStudio software v4.4.3. The multinomial model was implemented in R using the nnet::multinom() function.

### Ethics

This work has been approved by the Ethics Committee of the institution where the study was conducted (12/2022/CEFCM).

## RESULTS

### Characterization of the study sample

Patients’ demographic and clinical characteristics, overall and by previous biopsy status, are presented in Table-1. This study included 997 men, with a median age [P25; P75] of 68 [62; 72] years, from which 497 had undergone a previous biopsy that turned out negative. The median initial PSA and prostatic volume were 7.20 [5.04; 10.08] ng/mL and 57 [40; 79] mL, respectively. Both values were significantly higher in patients with previous biopsy (initial PSA: 8.26 [5.76; 11.24] ng/mL; prostatic volume: 64 [46; 87] mL) than in those biopsy-naïve (p<0.0001). Overall median PSA density was 0.12 [0.08; 0.19] ng/mL^2^ and more than half of the patients (62.5%) would be classified as being on low PCa risk group based on this parameter only.

**Table 1:** Demographic and clinical characterization of the study sample.

		Total (*n*=997)	No previous biopsy (*n*=496)*	Previous biopsy (*n*=497)*	P-value
**Age (years)**					0.0508
	Mean (SD)	66.90 (0.23)	66.30 (0.34)	67.45 (0.32)	
	Median [P25; P75]	68 [62; 72]	67 [61; 72]	68 [63; 72]	
	Min/Max	42/85	42/85	44/85	
**Initial PSA (ng/mL),**					**<0.0001**
	Mean (SD)	8.81 (0.33)	7.84 (0.54)	9.79 (0.39)	
	Median [P25; P75]	7.20 [5.04; 10.08]	6.19 [4.60; 8.41]	8.26 [5.76; 11.24]	
	Min/Max	0.50/253.00	0.50/253.00	0.53/150.00	
**Prostatic volume (mL)**					**<0.0001**
	Mean (SD)	63.83 (1.06)	57.50 (1.39)	70.17 (1.57)	
	Median [P25; P75]	57 [40; 79]	50 [38; 69]	64 [46; 87]	
	Min/Max	8/300	8/300	13/300	
**PSA density (ng/mL^2^)**					0.2279
	Mean (SD)	0.16 (0.00)	0.15 (0.01)	0.16 (0.01)	
	Median [P25; P75]	0.12 [0.08; 0.19]	0.12 [0.08; 0.18]	0.12 [0.08; 0.20]	
	Min/Max	0.01/2.14	0.01/2.14	0.01/1.56	
**PCa risk group based on PSA density,** *n* (%)					**0.8099**
	Low risk (≤0.15 ng/mL^2^)	618 (62.5%)	310 (62.9%)	305 (61.9%)	
	High risk (>0.15 ng/mL^2^)	371 (37.5%)	183 (37.1%)	188 (38.1%)	
	*Missing*	8	3	4	

* 4 patients had no information on previous biopsy status.

### Global and biopsy-specific PCa detection rate

A total of 282 (28.3%) patients were classified as PI-RADS 3, 534 (53.6%,) as PI-RADS 4 and 181 (18.1%) as PI-RADS 5 (Supplementary Figure-2). The description of the lesions’ location, size and histopathological features is presented in Supplementary Table-1 and Supplementary Table-2.

The overall rate of PCa positive cases detected by the combination approach (TB+SB) was 53.0% - 16.1% ciPCa and 36.8% csPCa (Table-2).

**Table 2:** PCa diagnosis by biopsy type.

		Total (n=997)			
		TB+SB	TB	SB	P-value
**PCa diagnosis**					
	Positive	528 (53.0%)	480 (48.1%)	194 (19.5%)	**<0.0010**
	Negative	469 (47.0%)	517 (51.9%)	803 (80.5%)	
**ISUP grade**					
	ciPCa (ISUP=1)	161 (16.1%)	133 (13.3%)	91 (9.1%)	
	csPCa (ISUP ≥2)	367 (36.8%)	347 (34.8%)	103 (10.3%)	
	No PCa	469 (47.0%)	517 (51.9%)	803 (80.5%)	
		Previous negative biopsy (n=497)			
**ISUP score**					
	ISUP 1	82 (16.5%)	65 (13.1%)	43 (8.7%)	**<0.0001**
	ISUP 2	64 (12.9%)	57 (11.5%)	27 (5.4%)	
	ISUP 3	46 (9.3%)	43 (8.7%)	14 (2.8%)	
	ISUP 4	31 (6.2%)	29 (5.8%)	5 (1.0%)	
	ISUP 5	9 (1.8%)	9 (1.8%)	1 (0.2%)	
	No PCa	265 (53.3%)	294 (59.1%)	407 (81.9%)	
**PCa**					
	ciPCa	82 (16.5%)	65 (13.1%)	43 (8.7%)	**<0.0001**
	csPCa	150 (30.2%)	138 (27.8%)	47 (9.5%)
	No PCa	265 (53.3%)	294 (59.1%)	407 (81.9%)

TB alone detected significantly more PCa cases than SB alone (48.1% vs. 19.5%, p<0.001).

The total csPCa cases diagnosed was three-fold higher with TB than with SB (34.8% vs. 10.3%; Table-2). Within each PI-RADS category, a similar result was observed, with TB detecting significantly higher csPCa rates (PI-RADS 3: p=0.021; PI-RADS 4: p<0.001; PI-RADS 5: p<0.001; Supplementary Table-3).

The comparison of PI-RADS and ISUP gradings by biopsy type are presented in Supplementary Tables 4 and 5, respectively. Analyzing PCa detection among patients with and without previous biopsy, the overall number of positive cases was higher for the biopsy-naïve group (59.3% vs. 46.9%, p<0.001; data not shown). This difference was observed particularly in the TB results (Supplementary Table-6), with this technique detecting significantly more positive cases in patients without previous biopsy than in those with a previous one (55.4% vs. 40.9%, p<0.001), namely csPCa, which was identified in nearly twice of the biopsy-naïve patients (41.7% vs. 27.8%, p<0.0001). On the contrary, in SB, a considerable smaller proportion of patients were diagnosed with PCa – 20.8% of biopsy-naïve patients and 18.2% of those who had already underwent a prostate biopsy (p=0.677).

Concerning patients with a previous negative biopsy (n=497), SB detected fewer PCa cases than TB (18.2% vs. 40.9%, p<0.0001), including csPCa (9.5% vs. 27.8%, p<0.0001), from 46.7% of total diagnosis (Table-2 and Supplementary Table-7). Still, adding SB to TB detected more 17 ciPCa and 12 csPCa cases, mainly ISUP 2 (Table-2, underlined) representing 12.5% of all PCa diagnosis in patients with a prior negative biopsy.

### Comparison between diagnosis and ISUP scoring following TB and SB

Overall, TB alone diagnosed more PCa cases and detected higher ISUP grades than SB. In fact, SB missed or underscored 369 (37.0%) cases of PCa, whereas TB missed or underscored only 53 (5.3%) PCa (Supplementary Table-8).

When considering ISUP grading (Table-3), TB underestimated 12 (1.2%) PCa cases and missed 48 (4.8%)- 34 (70.8%; 3.4% in the overall sample) ciPCa and 14 (29.2%; 1.4% in the overall sample) csPCA by SB. On the opposite, SB underscored 53 (5.3%) PCa cases and missed 334 (33.5%)—106 (31.7%; 10.6% in the overall sample) ciPCa and 228 (68.3%; 22.9% in the overall sample) csPCA by SB. Still, 576 (57.9%) cases were equally classified by both techniques (kappa coefficient=0.19; gray highlight).

**Table 3:** Comparison of ISUP grading between targeted and systematic biopsies. Relative frequencies relate to the total number of patients. Values in bold represent csPCa cases. Values below the gray shading indicate upgrading by TB, and values above gray shading indicate upgrading by SB.

	SB						
		No PCa (n=803)	ISUP 1 (n=91)	ISUP 2 (n=60)	ISUP 3 (n=26)	ISUP 4 (n=14)	ISUP 5 (n=3)
TB	No PCa (n=517)	469 (46.9%)	**34 (3.4%)**	**10 (1.0%)**	**3 (0.3%)**	**1 (0.1%)**	**0 (0.0%)**
	ISUP 1 (n=33)	**106 (10.6%)**	22 (2.2%)	**5 (0.5%)**	**0 (0.0%)**	**0 (0.0%)**	**0 (0.0%)**
	ISUP 2 (n=168)	**112 (11.2%)**	**20 (2.0%)**	**32 (3.2%)**	**2 (0.2%)**	**1 (0.1%)**	**1 (0.1%)**
	**ISUP 3 (n=93)**	**58 (5.8%)**	**7 (0.7%)**	**9 (0.9%)**	**16 (1.6%)**	**2 (0.2%)**	**1 (0.1%)**
	**ISUP 4 (n=66)**	**41 (4.1%)**	**7 (0.7%)**	**3 (0.3%)**	**5 (0.5%)**	**10 (1.0%)**	**0 (0.0%)**
	**ISUP 5 (n=20)**	**17 (1.7%)**	**1 (0.1%)**	**1 (0.1%)**	**0 (0.0%)**	**0 (0.0%)**	**1 (0.1%)**

kappa=0.19 (Slight agreement)

A detailed summary of differences in PCa diagnosis between TB and SB in positive patients is presented in Supplementary Table-9.

Overall, the SB method yielded a sensitivity of 36.7% and a specificity of 100.0%, corresponding to a number needed for diagnosis (NND) of 2.72 (95% CI, 2.46–3.06). In contrast, the TB method demonstrated a sensitivity of 90.9% with the same specificity of 100.0%, resulting in a significantly lower NND of 1.10 (95% CI, 1.07–1.13).

In a multinomial logistic regression using PCa cases detected by SB only (48 patients) as the reference, higher PSA (p=0.037) and larger prostate volume (p=0.036) were modestly associated with exclusive detection by TB (OR 1.05 per ng/mL PSA, 95% CI 1.00–1.09; OR 1.05 per mL prostate volume, 95% CI 1.00–1.10). Age and PSA density were not significantly associated with detection patterns.

## DISCUSSION

This retrospective single-center study provides new data on the diagnostic value of combining SB with TB.

The overall detection rate of PCa cases for combined biopsy (TB+SB) was 53.0%, of these 48.1% were diagnosed in TB and 19.6% in SB. These findings align with other studies (14, 15), where the detection of PCa following TB was 66% while for SB was 22% (15), as anticipated, as mpMRI enhances the detection of clinically significant disease (16). However, the diagnostic value of both techniques in scoring patients is still controversial. While one study showed lower ISUP grading with TB compared to SB – with 14% of the cases being upgraded in TB vs. 23% in SB, although non-significant (17), – other reported higher detection rates for ISUP scores ranging from 3 to 5. For instance, ISUP 4 was detected in 6.5% of SB cases vs. 10.2% for TB, and TB identified an additional 8.3% of cases above ISUP 3, compared to only 1.9% with SB (14). Accordingly, our results demonstrated the superiority of TB over SB in detecting ciPCa (13.3% vs. 9.1%) and csPCa cases (34.8% vs. 10.3%). CsPCa was defined as ISUP≥2. If ISUP 3 or higher were used, patients with intermediate-risk disease (considered clinically significant and therefore requiring treatment) would be excluded. Moreover, TB rarely underestimated the ISUP grading (12 cases, 1.2%) and missed only 4.8% of total PCa cases. This false negative rate can be explained by some small and less aggressive lesions being underestimated during imaging or by technical errors during imaging and biopsy (18). In fact, most cases (3.4%) were ciPCa and only 1.4% were csPCA by SB. A marginal addition of csPCa by SB may not justify the added cost of additional biopsies, patient discomfort and complications. Along with a global negative rate of 80.5% by SB – a higher value than that of other studies, which report from 14 to 37.6% of negative PCa (14, 19)– and the 33.5% false negative rate of the technique, these results support the limited value of the systematic approach in aiding to diagnosing PCa. Missed diagnoses in SB are often due to small tumors, anterior or apical lesions, and under sampling of heterogeneous cancers – limitations that are better addressed with TB (20, 21). Moreover, a general advantage of reducing the number of cores is a reduced number of complications like minor bleeding and urinary symptoms (22). Kalahati et al. have also found that undergoing SB is associated with a higher number of urinary infections (2.7% after SB and 1.7% after TB) (23). The difference in NND between the two approaches also indicated that fewer examinations are required with the TB method to achieve a correct diagnosis.

SB can miss csPCa, particularly in patients with ongoing suspicion after a negative result (20, 24). In fact, approximately half of this study sample had previously undergone a negative biopsy, with significantly increased PSA and prostatic volume. This suggests a population that likely already presented risk factors prompting this first biopsy. More importantly, the rate of PCa detection was higher with TB, compared to SB in these patients (40.9% vs. 18.1%, from 46.7% total cases), especially csPCa, which supports the notion that there is no added value in performing a SB combined with TB after a negative SB result. PCa detection by TB also differed significantly between patients with and without a previous biopsy (40.9% vs. 55.2%). The high predictive value of TB for accurately determining a patient's true pathological grade group reduces the risk of misdiagnosis and may decrease diagnostic uncertainty, supporting a TB-only strategy in men with previous negative biopsies, alike reported before (21, 25). Additionally, our results support the clinical utility of TB particularly in patients with higher PSA levels or larger prostate volumes, likely reflecting sampling dilution effects inherent to biopsy procedures in larger glands. On the other hand, omitting SB could result in missed ciPCa, which are typically slow-growing and less aggressive tumors but may still pose long-term risks. However, high-risk patients can benefit from active surveillance, enabling timely detection of disease progression and early intervention. Collectively, our findings contribute by showing that undergoing TB alone is sufficient for many patients.

This study has several strengths, namely, including a high number of participants and biopsy samples, enrolled both biopsy-naïve patients and those with previous biopsy, and used a mapping grid with predetermined holes, to minimize of oversampling risk and targeting bias. All biopsies were performed by the same experienced urologist, thus minimizing operator-dependent variability and ensuring consistent, standardized assessments. Limitations of this study include the single-center design, which may restrict the generalizability of the findings, and the absence of whole mount prostatectomy for histopathological verification. Future studies are needed to develop risk-adapted models incorporating mpMRI findings, PSA and clinical parameters to determine when SB can be safely omitted. Long-term follow-up is also needed to evaluate the clinical impact of missed csPCa cases in patients undergoing TB alone.

## CONCLUSIONS

**Figure 1:**
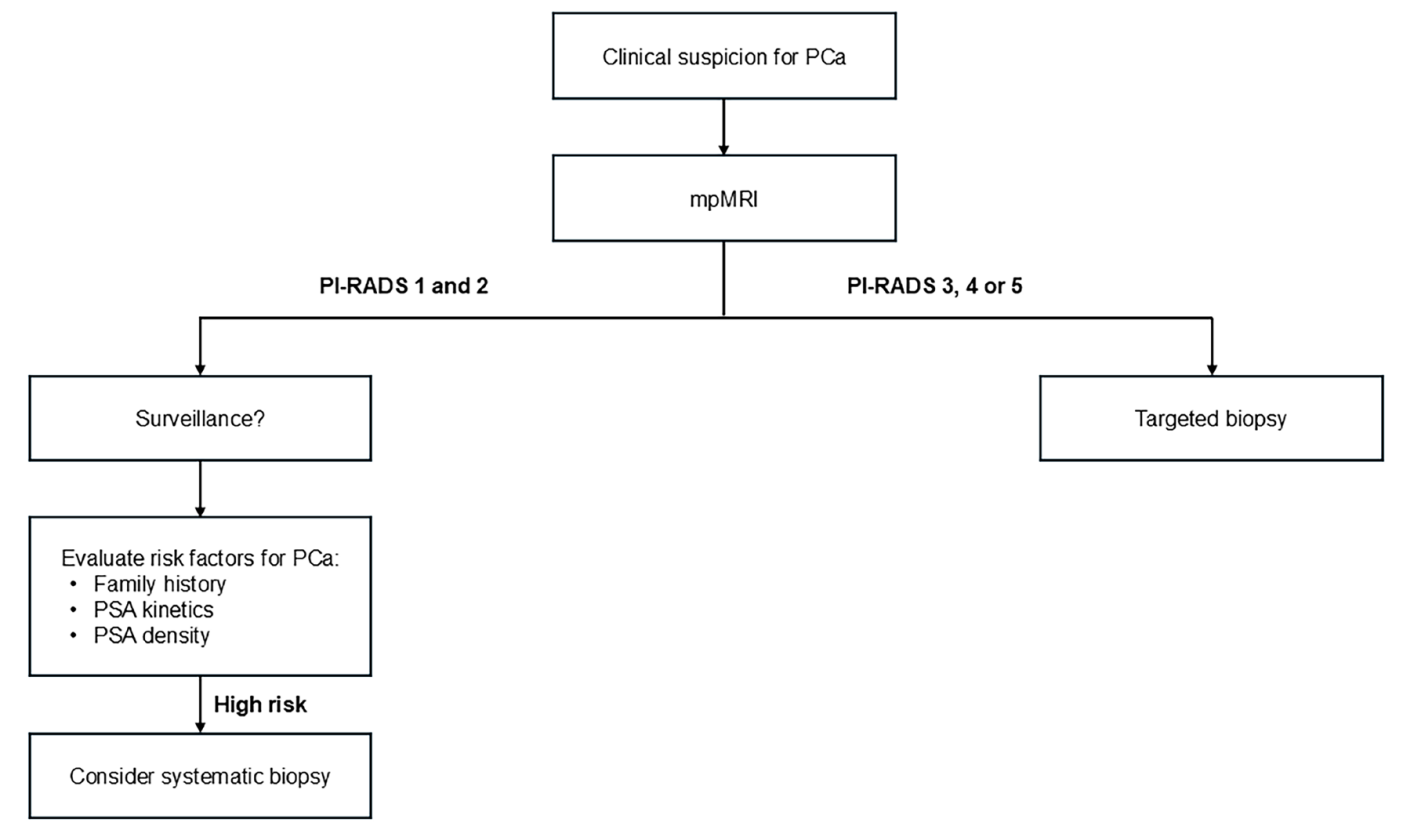
Clinical algorithm flowchart for PCa biopsy.

Overall, these results suggest that SB offers limited diagnostic value when combined with a mpMRI-targeted approach and support the latter as a stand-alone procedure in men with suspicious lesions (Figure-1). Omitting SB would miss few PCa cases and result in marginal underdiagnosis of csPCa, particularly in previously biopsied patients. This less invasive approach could enhance patient comfort, streamline clinical decision-making, and enhance clinical outcomes.

## Data Availability

Data will be available upon request
