# Supplementary material for: Is there still a role for systematic biopsy after targeted biopsy for the detection of clinically significant prostate cancer in MRI suspicious lesions?
Source: Int Braz J Urol. 2026 Feb 28;52(3):e20250653. doi: 10.1590/S1677-5538.IBJU.2025.0653 (PMC13124187; doi:10.1590/S1677-5538.IBJU.2025.0653)
Supplement: APPENDIX [file 1677-6119-ibju-52-03-e20250653-suppl1.pdf]

APPENDIX

Supplementary Figure 1 - Examples of four sample grid mappings.

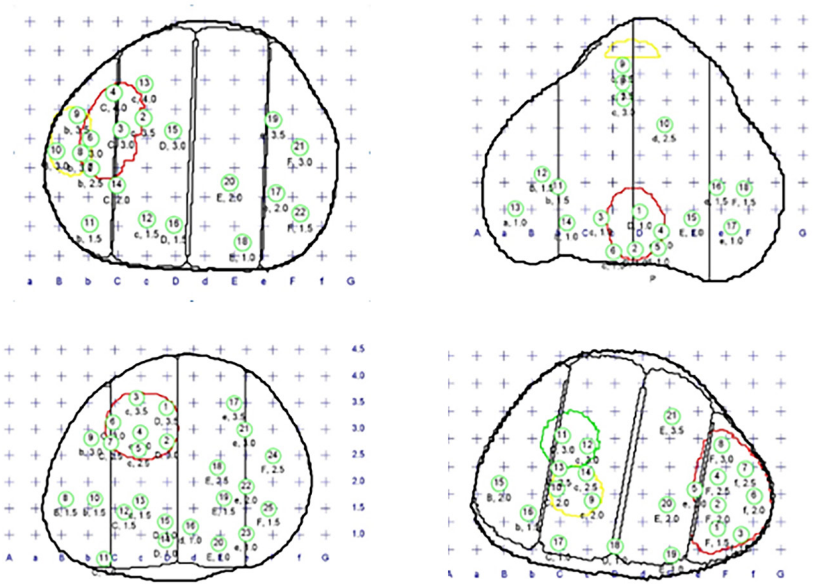

Supplementary Figure 2 - PI-RADS scoring of patients. Data are shown as absolute and relative frequencies.

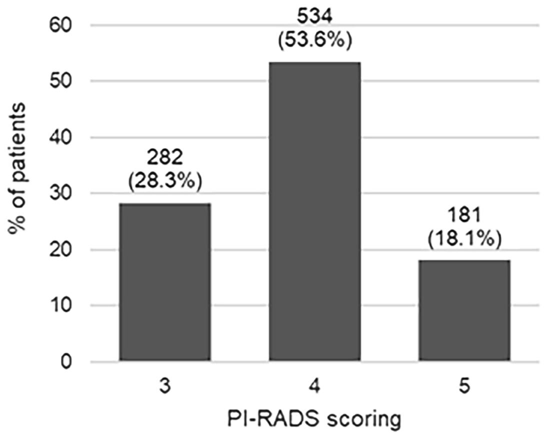

Supplementary Table 1 - Lesions characterization following mpMRI.

	n=997
**Lesion location**	
**By zonal anatomy,** *n* **(%)**	
Central	267 (26.8)
Central/Peripheric	4 (0.4)
Peripheric	726 (72.8)
**By prostate region,** *n* **(%)**	
Apex	248 (25.0)
Base	204 (20.5)
Base/Middle	45 (4.5)
Base/Middle/Apex	6 (0.6)
Middle	436 (43.9)
Middle/Apex	55 (5.5)
Missing	3
**Lesion size (mm)**	
Median [P25; P75]	10.0 [8.0; 14.0]
Min / Max	1.0 / 49.0

Supplementary Table 2 - Histopathological features of PCa lesions.

	Total (***n***=528)	No previous biopsy (***n***=294)	Previous biopsy (***n***=232)
**Cribriform pattern, *n (%)***			
Yes	37 (7.1)	24 (8.3)	13 (5.6)
No	487 (92.9)	266 (91.7)	219 (94.4)
Missing	4	4	0
**Lymphovascular invasion, *n* (%)**			
Yes	1 (0.2)	0 (0.0)	1 (0.4)
No	524 (99.8)	291 (100.0)	231 (99.6)
Missing	3	3	0
**Perineural invasion, *n* (%)**			
Yes	117 (22.3)	78 (26.8)	39 (16.8)
No	408 (77.7)	213 (73.2)	193 (83.2)
Missing	3	3	0

Supplementary Table 3 - PI-RADS grading by biopsy approach.

		TB (n=997)		SB (n=997)	
		PCa	csPCa (ISUP ≥2)	PCa	csPCa (ISUP ≥2)
PI-RADS score	**3 (*n*=282)**	68 (24.1%)	35 (12.4%)	43 (15.2%)	18 (6.4%)
	**4 (*n*=534)**	272 (50.9%)	195 (36.5%)	113 (21.2%)	61 (11.4%)
	**5 (*n*=181)**	140 (77.3%)	117 (64.6%)	38 (21.0%)	24 (13.3%)

csPCa = PI-RADS 3; $X^2 = 5.33$; p=0.021; PI-RADS 4: X^2=90.88; p<0.001; PI-RADS 5: X^2=104.65, p<0.001

Supplementary Table 4 - Comparison between PI-RADS and ISUP grading after targeted biopsy. Relative frequencies relate to the number of PI-RADS patients in each category.

**TB (n=997)**		ISUP					
		No PCa (n=518)	ISUP 1 (n=132)	ISUP 2 (n=169)	ISUP 3 (n=93)	ISUP 4 (n=66)	ISUP 5 (n=20)
**PI-RADS**	**3 (n=282)**	214 (75.9%)	33 (11.7%)	24 (8.5%)	9 (3.2%)	2 (0.7%)	0 (0.0%)
	**4 (n=534)**	262 (49.1%)	77 (14.4%)	94 (17.6%)	53 (9.9%)	40 (7.5%)	8 (1.5%)
	**5 (n=181)**	41 (22.7%)	23 (17.7%)	50 (27.6%)	31 (17.1%)	24 (13.3%)	12 (6.6%)

Supplementary Table 5 - Comparison between PI-RADS and ISUP grading after systematic biopsy. Relative frequencies relate to the number of PI-RADS patients in each category.

**SB (n=997)**		ISUP					
		No PCa (n=803)	ISUP 1 (n=91)	ISUP 2 (n=61)	ISUP 3 (n=26)	ISUP 4 (n=14)	ISUP 5 (n=3)
**PI-RADS**	**3 (n=282)**	239 (84.8%)	25 (8.9%)	12 (4.3%)	4 (1.4%)	1 (0.3%)	1 (0.3%)
	**4 (n=534)**	421 (78.8%)	52 (9.7%)	35 (6.6%)	15 (2.8%)	9 (1.7%)	2 (0.4%)
	**5 (n=181)**	143 (79.0%)	14 (7.7%)	13 (7.2%)	7 (3.9%)	4 (2.2%)	0 (0.0%)

Supplementary Table 6 - ISUP grading after targeted and systematic biopsy, by prior biopsy status.

	TB			SB		
	No previous biopsy (n=496)	Previous biopsy (n=497)	P-value	No previous biopsy (n=496)	Previous biopsy (n=497)	P-value
**ISUP score, n (%)**						
ISUP 1	68 (13.7%)	65 (13.1%)		48 (9.7%)	43 (8.7%)	
ISUP 2	110 (22.1%)	57 (11.5%)		33 (6.7%)	27 (5.4%)	
ISUP 3	50 (10.1%)	43 (8.7%)	**<0.0001**	12 (2.4%)	14 (2.8%)	0.813
ISUP 4	36 (7.3%)	29 (5.8%)		8 (1.6%)	5 (1.0%)	
ISUP 5	11 (2.2%)	9 (1.8%)		2 (0.4%)	1 (0.2%)	
No PCa	221 (44.6%)	294 (59.1%)		393 (79.2%)	407 (81.9%)	
**Significant PCa?, n (%)**						
ciPCa (ISUP=1)	68 (13.7%)	65 (13.1%)		48 (9.7%)	43 (8.7%)	
csPCa (ISUP ≥2)	207 (41.7%)	138 (27.8%)	**<0.0001**	55 (11.1%)	47 (9.5%)	0.677
No PCa	221 (44.6%)	294 (59.1%)		393 (79.2%)	407 (81.9%)	

Supplementary Table 7 - PCa diagnosis and ISUP grading in patients with previous biopsy.

	Previous negative biopsy (n=497)			
	TB+SB	TB	SB	p-value
**ISUP score**				
ISUP 1	82 (16.5%)	65 (13.1%)	43 (8.7%)	
ISUP 2	64 (12.9%)	57 (11.5%)	27 (5.4%)	
ISUP 3	46 (9.3%)	43 (8.7%)	14 (2.8%)	**<0.0001**
ISUP 4	31 (6.2%)	29 (5.8%)	5 (1.0%)	
ISUP 5	9 (1.8%)	9 (1.8%)	1 (0.2%)	
No PCa	265 (53.3%)	294 (59.1%)	407 (81.9%)	
**PCa**				
ciPCa	82 (16.5%)	65 (13.1%)	43 (8.7%)	**<0.0001**
csPCa	150 (30.2%)	138 (27.8%)	47 (9.5%)	
No PCa	265 (53.3%)	294 (59.1%)	407 (81.9%)	

Supplementary Table 8 - Comparison of the PCa clinical significance between targeted fusion and systematic biopsy. Relative frequencies relate to the total number of patients. Values in bold represent csPCa cases. Values above gray shading indicates upgrading by TB, values below gray shading indicate upgrading by SB.

		SB		
		No PCa (n=803)	ciPCa (n=91)	csPCa (n=103)
**TB**	No PCa (n=517)	469 (46.9%)	34 (3.4%)	**14 (1.4%)**
	ciPCa (n=133)	106 (10.6%)	22 (2.2%)	**5 (0.5%)**
	csPCa (n=347)	**228 (22.9%)**	**35 (3.5%)**	**84 (8.4%)**

Supplementary Table 9 - Comparison of PCa diagnosis between targeted and systematic biopsies in patients with PCa positive fragments in at least one biopsy method.

	**PCa positive patients (*n*=528)**
**PCa diagnosis between biopsy types**	
**Negative TB and positive SB**	**48 (9.1%)**
ISUP 1 (SB)	34 (6.4%)
ISUP 2 (SB)	10 (1.9%)
ISUP 3 (SB)	3 (0.6%)
ISUP 4 (SB)	1 (0.2%)
ISUP 5 (SB)	0 (0.0%)
**Positive TB and negative SB**	**334 (63.3%)**
**Positive TB, but less aggressive than positive SB**	**12 (2.3%)**
ISUP 1 (TB) vs ISUP 2 (SB)	5 (0.9%)
ISUP 2 (TB) vs ISUP 3/4/5 (SB)	4 (0.8%)
ISUP 3 (TB) vs ISUP 4/5 (SB)	3 (0.6%)
ISUP 4 (TB) vs ISUP 5 (SB)	0 (0.0%)
**Positive TB, with the same or greater aggressiveness than positive SB**	**134 (25.4%)**
